# Designing micro- and mesoporous carbon networks by chemical activation of organic resins

**DOI:** 10.1007/s10450-016-9851-4

**Published:** 2016-12-15

**Authors:** Alicia Gomis-Berenguer, Raquel García-González, Ana S. Mestre, Conchi O. Ania

**Affiliations:** 1 ADPOR Group, Instituto Nacional del Carbon (INCAR, CSIC), 33011 Oviedo, Spain; 2 Centro de Química e Bioquímica, Faculdade de Ciências, Universidade de Lisboa, 1749-016 Lisboa, Portugal

**Keywords:** Mesoporous polymeric resins, Carbon xerogels, Chemical activation, Impregnation methodology, Textural characterization

## Abstract

Carbon xerogels with ultrahigh micro- and mesopore volumes were synthesized from the activation of polymeric resins prepared by sol–gel polycondensation of resorcinol/formaldehyde mixtures in basic medium and subcritical drying. Various activating conditions (e.g., agent, temperature, impregnation conditions) were used and it was found that the textural features of the resulting carbon xerogels are linked to the experimental procedure of the activation reaction to promote the porosity development. The shrinkage and structural collapse of the fragile resins typically obtained upon annealing at high temperatures (during carbonization and/or physical activation) is suppressed when the impregnation of the chemical activating agent is performed under controlled conditions. If the alkaline reagent (either KOH or K_2_CO_3_) is put in contact with the resin by wet impregnation (liquid/solid); under such conditions, the intimate contact between both compounds allows the formation of microporosity during the activation along with the enlargement and/or preservation of the mesoporosity of the pristine resin. Furthermore, the chemical activation via wet impregnation allows the combination of high surface areas and the preservation (even higher development) of the mesoporosity created during the synthesis of the resin. The effect of the impregnation method was found highly dependent of the reagent and activation temperature, highlighting the possibility to design micro-mesoporous carbon xerogels at low temperatures with a subtle control of the activation conditions.

**Electronic supplementary material:**

The online version of this article (doi:10.1007/s10450-016-9851-4) contains supplementary material, which is available to authorized users.

## Introduction

The preparation of nanoporous carbons with high surface areas and pore volumes with tailor-made distribution of pore sizes within the full micro-/mesoporous range has become a subject of great interest, driven by the need to obtain high performing carbons in multidisciplinary fields. Porous carbon materials normally have relatively broad pore-size distributions in both micropore and mesopore ranges, being the porosity dominated by the micropore structure (Marsh and Rodriguez-Reinsono 2006; Wang and Kaskel 2012).

Despite the wide applications of nanoporous carbons in adsorption, separation and catalysis, very often they suffer from limitations associated to slow mass transfer due to the small dimensions of the nanopores. The combination of large specific surface areas (microporosity) with an adequate network of transport pores is desirable to favor diffusion and/or adsorption of bulky molecules of strategic interest in environmental remediation (e.g., hormones, pharmaceuticals, dyes), energy storage and conversion (e.g. electrocatalysis, supercapacitors) and sensing applications (e.g., immobilization of enzymes) (Xin and Song 2015; Liang et al. 2008; Pröbstle et al. 2002; Rasines et al. 2012).

In this context, the synthesis of carbon gels is an interesting approach for the preparation of highly porous materials with controlled properties due to their relatively low-cost and unique physical, chemical and electrochemical properties (Al-Muhtaseb and Ritter 2003; Elkhatat and Al-Muhtaseb 2011; Job et al. 2004; Fellinger et al. 2012). The synthesis of carbon gels is typically based on the sol–gel polycondensation of resorcinol and formaldehyde (RF) mixtures in the presence of a catalyst (Pekala 1989). The general mechanism depends on the solution pH, the components molar ratios, temperature, and drying conditions among other parameters (Al-Muhtaseb and Ritter 2003; Lin and Ritter 1997; Czakkel et al. 2005). The organic RF gels are eventually carbonized to obtain a thermally stable carbon gel.

Although the main properties of the RF gel may be tuned during the synthesis and drying process, the final characteristics of the material can be strongly modified by the carbonization process and/or further activation, often leading to a partial collapse of the porosity of the carbon gel. This is particularly critical in xerogels prepared by a subcritical drying that suffer from low mechanical resistance (Al-Muhtaseb and Ritter 2003). As opposed to carbon aerogels prepared by supercritical drying (Pekala 1989), the fragile polymeric structure of the xerogels usually undergoes an outstanding shrinkage and/or structural collapse during the carbonization step to obtain more dense carbon gels. Further activation of the RF xerogel to develop the nanoporous structure exerts a similar effect; whereas the microporosity is easily tuned by the activation procedure, the mesoporosity of the polymeric organic resins is typically destroyed during the activation process, due to the above-mentioned fragile structure of the xerogel dried under subcritical conditions (Zubizarreta et al. 2008; Conceição et al. 2009; Lozano-Castelló et al. 2002; Contreras et al. 2010; Isaacs-Paez et al. 2015; Hayashi et al. 2002; Macias et al. 2013, 2016).

We herein report the activation of organic polymeric resins dried under subcritical conditions (organic xerogels) to obtain highly nanoporous carbon xerogels with large microporosity and preserving the unique mesoporous network of the pristine resins. The porous features of the resulting carbon xerogels have been exhaustively characterized by high resolution gas adsorption isotherms with several probes at various temperatures. Data has shown different trends on the porosity of the carbons depending on the chemical agent (potassium carbonate vs. hydroxide) and the impregnation procedure, leading to an important development of both the micro- and mesoporosity of the carbon material, as opposed to the porosity of the carbon obtained from the classical physical activation under CO_2_ of the same precursor. The resulting carbon xerogels materials exhibit an unusual bimodal distribution of pore sizes within the micro and mesopore range, anticipating an outstanding performance of these materials in applications where adsorption capacity and fast mass transfer are required.

## Experimental

### Materials synthesis

Nanoporous resorcinol/formaldehyde polymeric resins were synthesized by the sol–gel polymerization of resorcinol (R) and formaldehyde (F) in water (W), using sodium carbonate (C) as catalyst, as reported elsewhere (Haro et al. 2011). Briefly, the precursors (molar ratios R/C 200, R/W 0.06 and R/F 0.5) were mixed under magnetic stirring and immediately heated in airtight sealed glass vessels for gelation/aging at 95 °C for 4 h in an oven. Afterwards, the wet gels were dried at subcritical conditions at 150 °C for 12 h without solvent removal. The obtained RF resin was labeled as OG. Sample OG was further activated under different conditions (i.e., temperature, activating agent). For the physical activation, the pristine OG resin was initially treated at 800 °C under inert atmosphere (i.e., 100 mL/min N_2_) for 1 h (sample OG-py) and then gasified using CO_2_ (10 mL/min) at the same temperature and at different burn-off degrees (samples OG-phys-Q, where Q stands for the burn-off degree). For the chemical activation in K_2_CO_3_ and KOH, a molar ratio resin:activating agent of 1:1 was fixed. The chemical activation was carried out under inert atmosphere (300 mL/min N_2_, 1 h) up to various temperatures ranging from 600 to 800 °C. Subsequently, the samples were thoroughly washed with hot water (until constant pH). Two impregnation routes were used to allow the contact between the chemical reactant and the resin powders: (i) physical mixing of both powders (P) in a mortar and (ii) wet impregnation (W) by dissolving the activating agent in ca. 20 mL of water, and stirring for 24 h at room temperature. In the latter case the suspension is dried at 80 °C before carbonization, to allow the evaporation of the excess water. The chemically activated samples were labeled as OGX-Y-Z where X refers to the activation temperature (i.e., 6, 7 or 8 for 600, 700 and 800 °C, respectively), Y refers to the activating agent (KC for K_2_CO_3_ and KO for KOH), and Z refers to the impregnation procedure (P for physical mixing and W for wet impregnation). For instance, sample OG8-KC-W is the resin chemically activated at 800 °C using K_2_CO_3_ by wet impregnation.

### Gas adsorption

The porosity of the samples was characterized by high resolution equilibrium adsorption–desorption isotherms of various gases (N_2_ and CO_2_ at −196 and 0 °C, respectively) measured in a volumetric analyzer (Micromeritics). The instrument was equipped with a molecular drag vacuum pump and three pressure transducers (0.1, 10, 1000 Torr, uncertainty within 0.15% of each reading) to enhance the sensitivity in the low-pressure range. Before the analysis, the samples were degassed under dynamic vacuum (ca. 10^−5^ Torr) at 120 °C for 17 h. Strict analysis conditions were programmed during the gas adsorption measurements to ensure equilibrium data, thus the average elapsed time for each isotherm was 90–120 h. Each isotherm measurement was performed in duplicate to guarantee the accuracy of the experiments (error was below 0.1%) and to obtain reproducible data. All the gases were supplied by Air Products with ultrahigh purity (i.e., 99.995%). The isotherms were used to calculate the specific surface area using the Brunauer–Emmett–Teller theory, S_BET_, and the total pore volume, V_TOTAL_. The PSD analysis in the full micro-mesopore range was calculated using the 2D-NLDFT-HS model assuming surface heterogeneity of pores (Jagiello and Olivier 2013). The narrow microporosity was further assessed by CO_2_ adsorption isotherms at 0 °C using the Dubinin-Radushkevich (DR) equation.

### Scanning electron microscopy (SEM)

SEM images were performed at Zeiss Supra 55 VP. The accelerating voltage was 5.00 kV. Scanning was performed in situ on a sample powder without coating.

## Results and discussion

### Physical vs. chemical activation

**Fig. 1:**
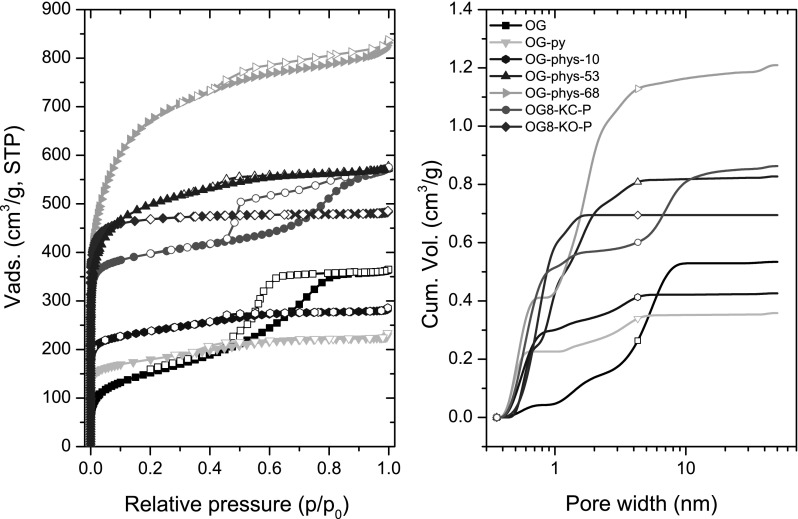
**a** High resolution N_2_ adsorption (*solid symbols*) and desorption (*open symbols*) isotherms at −196 °C of the pristine resin and the carbon xerogels after carbonization and activation at 800 °C using CO_2_, K_2_CO_3_ and KOH; **b** Cumulative pore size distributions obtained from the 2D-NLDFT-HS method applied to the N_2_ adsorption isotherms

**Fig. 2:**
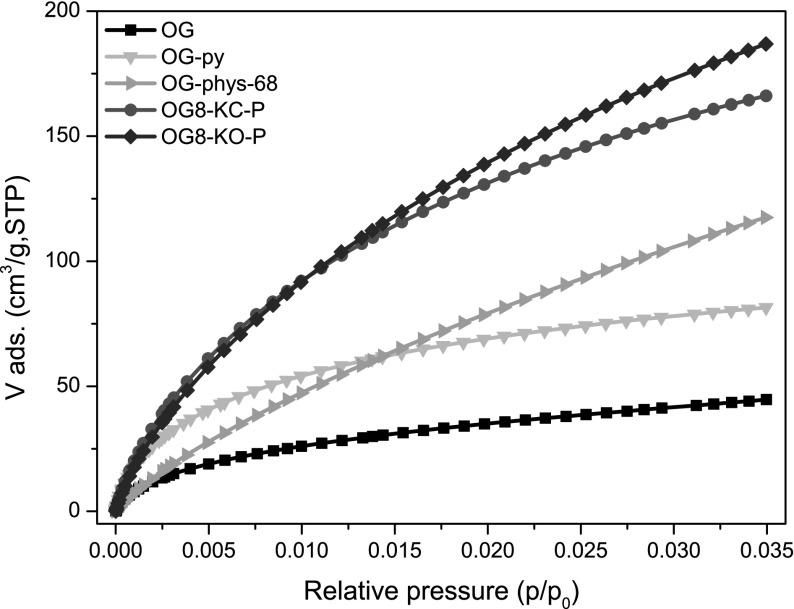
CO_2_ adsorption isotherms at 0 °C of the pristine resin and the carbon xerogels after carbonization and activation at 800 °C using CO_2_, K_2_CO_3_ and KOH

Figure 1 shows the N_2_ adsorption isotherms at −196 °C of the pristine resin and the carbon xerogels obtained by physical activation in CO_2_ and conventional chemical activation at 800 °C (i.e., physical mixture). The porosity of the organic resin (sample OG) displays a type IVa isotherm with a prominent H2 hysteresis loop in the desorption branch at relative pressures ~0.4–0.8, characteristic of micro/mesoporous solids. The main textural parameters (Table 1) are in agreement with those reported for RF polymeric organic gels prepared using high R/C ratio (Al-Muhtaseb and Ritter 2003; Macias et al. 2013). Data shows that the porosity of OG resin is mainly composed of mesopores, with a small contribution of micropores, as also corroborated by the analysis of the narrow microporosity from the CO_2_ adsorption data (Fig. 2). The carbonization at 800 °C (sample OG-py) provoked important changes in the shape of the adsorption–desorption isotherm, with an increased gas uptake at low relative pressures (micropore region) and a sharp fall in the mesopore volume (from 0.4 to 0.08 cm^3^/g) that was accompanied by a downward shift in the position of the hysteresis loop, indicating the shrinkage in the average mesopore size. These changes are attributed to the shrinkage of the xerogels as a result of the densification of the matrix upon carbonization (leading to the formation of microporosity within the nodules), resulting in the collapse of the voids between the spherical particles that are responsible for the mesoporosity of the sample. This contrasts with the trend reported for organic gels dried under supercritical conditions, where the carbonization increases the microporosity while preserving the mesopore structure (Al-Muhtaseb and Ritter 2003; Rasines et al. 2015). The carbonization treatment yielded a material with a higher micropore volume (sample OG-py) than the pristine resin (Fig. 2), due to the formation of narrow pores upon the thermal decomposition of the resin (Fig. S1).

**Table 1:** Porosity parameters obtained from N_2_ adsorption isotherms at −196 °C for the pristine resin and the carbon xerogels after carbonization, physical activation at 800 °C under CO_2_ and chemical activation using K_2_CO_3_ and KOH at 800 °C

	Yield^b^ (%)	S_BET_ (m^2^/g)	V_TOTAL_^c^ (cm^3^/g)	V_micro_^d^ (cm^3^/g)	V_meso_^d^ (cm^3^/g)
OG	–	552	0.56	0.14	0.40
OG-py	45^a^	682	0.38	0.24	0.08
OG-phys-10	41	920	0.43	0.35	0.08
OG-phys-53	21	1898	0.88	0.70	0.13
OG-phys-68	14	2384	1.27	0.91	0.30
OG8-KC-P	24	1644	0.97	0.56	0.32
OG8-KO-P	21	1904	0.75	0.68	n.d.

*n.d*. not detected

^a^Carbonization yield in the case of OG-py

^b^Yield after activation vs. the precursor sample OG

^c^Measured at ~0.99 of relative pressure

^d^Measured applying the 2D-NLDFT-HS method

Further activation under CO_2_ atmosphere (samples OG-phys-Q) resulted in the development of the microporous structure (surface areas up to three times larger than for the pristine or carbonized resin), whereas the mesoporosity of the pristine resin is lost. For all the burn-off degrees, the samples present type I N_2_ adsorption isotherms (Thommes et al. 2015) with almost negligible hysteresis loops, indicating the enlargement of the micropores after the activation and a small contribution of narrow mesopores. These are the typical porous features obtained for other carbon precursors after activation for long times (Marsh and Rodriguez-Reinoso 2006).

In the case of the chemical activation (samples OG-KO-P and OG-KC-P), the process is more complex as the organic xerogel undergoes thermal decomposition simultaneously to the activation reaction, likely leading to a higher reactivity given the chemical composition of the resin (rich in labile groups, Fig. S1). Interestingly, the extent of the activation (in terms of porous development) was heavily dependent on the nature of the activating agent, although similar yields were obtained in both cases (Table 1). The activation using KOH (sample OG8-KO-P) resulted in a high development of the microporosity-mainly narrow micropores of small sizes (Fig. 2), in agreement with the numerous works in the literature reporting the chemical activation of various precursors (Lillo-Ródenas et al. 2004; Lozano-Castelló et al. 2001; Molina-Sabio et al. 2004; Marsh and Rodriguez-Reinoso 2006 and references therein). In contrast, the mesoporosity of the organic resin disappeared, as inferred from the almost negligible hysteresis loop in the desorption branch of the N_2_ adsorption isotherm.

On the other hand, sample OG8-KC-P displayed a type IVa isotherm, characteristic of a bimodal micro-mesopore system, with a prominent H2(a) hysteresis loop and a well-defined knee in the isotherm at low relative pressures (indicating the development of microporosity). Furthermore, the hysteresis loop of the isotherm expanded to a wider range of relative pressures (from 0.4 to 0.9), indicating the existence of a complex system of heterogeneous mesoporosity comprised of pores of different widths. Indeed, the hysteresis loop is smooth in the adsorption branch but shows a steep desorption branch, indicating the presence of narrow pore necks and pore blocking effects in the mesoporosity (Thommes et al. 2015). Despite their differences in the shape of the N_2_ adsorption–desorption isotherms, both samples OG8-KO-P and OG8-KC-P displayed quite similar total and micropore volumes (Table 1), with differences mostly affecting the mesoporosity.

The analysis of the pore size distribution of the samples (Fig. 1b; Fig. S2) confirmed the shrinkage of the mesoporous structure upon carbonization and physical activation, with the average mesopore size decreasing from ca. 5–6 nm for OG to values of about 2–3 nm for OG-py and OG-phys-Q, respectively. In contrast, sample OG8-KC-P displayed a bimodal micro-mesopore system with a wide mesoporosity comprised of small and large mesopores (centered at about 7 nm). Thus, the chemical activation reaction with K_2_CO_3_ prevented the collapse of the porosity of the organic xerogel induced by the effect of the thermal treatment at high temperature, leading to the formation of new microporosity accompanied by the enlargement of the mesopore structure. On the other hand, sample OG8-KO-P displayed a distribution of pore sizes similar to that of the samples obtained by physical activation, with a large fraction of micropores and an almost negligible volume of mesopores (Fig. 1b). As for the narrow microporosity, the samples obtained by chemical activation presented higher volumes of CO_2_ adsorbed (Fig. 2) than those obtained by physical activation, indicating the existence of pores of smaller sizes. Interestingly, despite their differences in the N_2_ adsorption isotherms, samples OG8-KC-P and OG8-KO-P presented similar narrow micropore volumes (Table 2). Also, the less pronounced curvature of the CO_2_ adsorption isotherm of sample OG8-phys-68 at low relative pressures (compared to the samples prepared by chemical activation) indicates the occurrence of pores of larger dimensions (in average).

**Table 2:** Porosity parameters obtained from N_2_ and CO_2_ adsorption isotherms at −196 and 0 °C, respectively, of the chemically activated xerogels using K_2_CO_3_ and KOH at various temperatures and impregnation methods

	Yield (%)	S_BET_ (m^2^/g)	V_TOTAL_ (cm^3^/g)^a^	V_micro_ (cm^3^/g)^b^	V_meso_ (cm^3^/g)^b^	W_0_ CO_2_ (cm^3^/g)^c^	Ratio V_micro_/V_meso_
OG	–	552	0.56	0.14	0.40	0.13	0.35
OG6-KC-P	34	811	0.51	0.31	0.21	0.36	1.48
OG6-KC-W	34	875	0.68	0.31	0.37	0.35	0.84
OG6-KO-P	42	914	0.40	0.35	0.04	0.37	8.75
OG6-KO-W	39	841	0.82	0.29	0.53	0.31	0.55
OG7-KC-P	51	848	0.43	0.35	0.10	0.39	3.50
OG7-KC-W	44	1304	0.85	0.43	0.38	0.48	1.13
OG7-KO-P	49	1160	0.79	0.45	0.17	0.49	2.65
OG7-KO-W	48	1341	0.89	0.52	0.36	0.49	1.44
OG8-KC-P	24	1576	0.88	0.56	0.32	0.55	1.75
OG8-KC-W	23	1644	0.97	0.54	0.30	0.53	1.80
OG8-KO-P	24	1904	0.75	0.60	n.d.	0.66	–
OG8-KO-W	23	1756	0.69	0.66	0.01	0.70	66

*n.d*. not detected

^a^Total pore volume evaluated at p/p_0_ ~0.99 from the N_2_ adsorption isohterms

^b^Evaluated from 2D-NLDFT-HS applied to the N_2_ adsorption isohterms

^c^Evaluated from DR method applied to the CO_2_ adsorption isohterms

Other studies on the activation of RF gels from the literature (Calvo et al. 2011; Zubizarreta et al. 2008; Job et al. 2005; Rasines et al. 2015; Macias et al. 2013) have reported differences in the porosity of the resulting materials depending on the composition of the gels. For carbon aerogels, it has been reported that it is possible to increase the microporosity upon activation without a significant modification of the meso/macroporosity of the precursor (depending on the synthesis conditions of the aerogel). However, the activation of xerogels typically provokes the loss of the meso/macroporosity. These results contrast with herein reported data, as the mesoporosity of the precursor is inherited by the samples activated using K_2_CO_3_, but not in the case of KOH (these alkaline hydroxides are commonly used in the literature), which may be linked to the fact that KOH is more effective than K_2_CO_3_ for activation (Wang and Kaskel 2012; Mestre et al. 2014, 2015). To further understand this behavior, we have explored the effect of the impregnation procedure (physical mixing and wet impregnation) for both activating agents, aiming to design a simple approach for the synthesis of highly nanoporous carbon gels with bimodal micro/mesoporous structure.

### Chemical activation: effect of the impregnation procedure and activation temperature

**Fig. 3:**
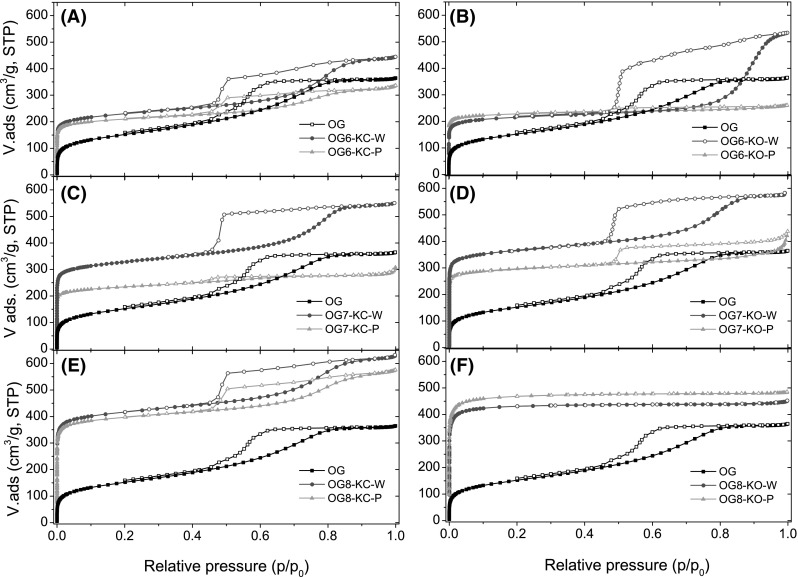
Adsorption (*solid symbols*) and desorption (*open symbols*) isotherms of N_2_ at −196 °C of all activated gels with K_2_CO_3_ and KOH by both, physical (P) and wet (W) impregnation at different temperatures (600, 700 and 800 °C)

Figure 3 and S3 show the N_2_ adsorption isotherms at −196 °C of the samples prepared using different impregnation procedures (W and P); the textural parameters are presented in Table 2. As seen, substantial differences are obtained on the porosity of the samples depending on the activation conditions (in terms of temperature, impregnation method and activating agent).

**Fig. 4:**
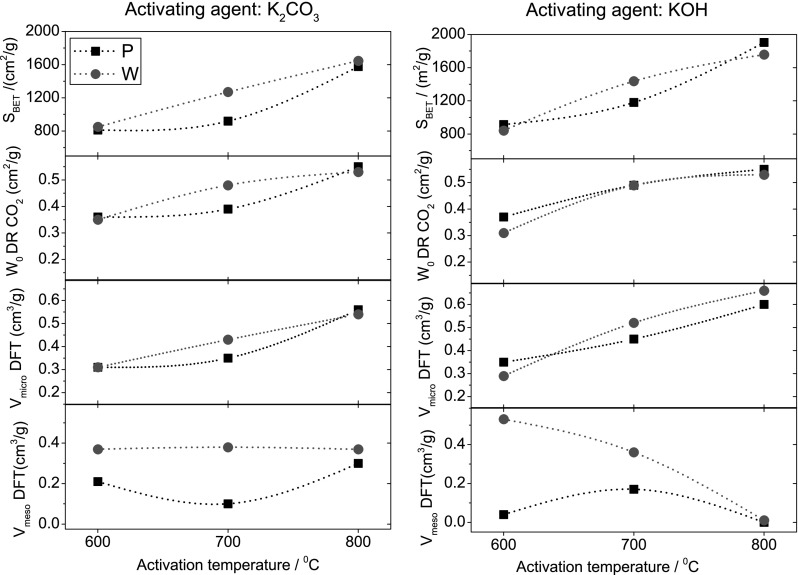
Correlation between the main textural parameters (specific surface area, micropore and mesopore volumes) of activated xerogels as a function of activated temperature with (**a**) potassium carbonate and (**b**) potassium hydroxide for both impregnation methods

Regarding temperature, the reactivity profiles of the organic xerogel (Fig. S4) revealed that the activation reaction in both cases starts at relatively low temperatures for all the mixtures (lower that those corresponding to the decomposition of K_2_CO_3_ and KOH themselves); thus 600, 700 and 800 °C were chosen to evaluate the effect of the activation temperature. According to literature, the development of the porosity is favored at high activation temperatures, reaching a maximum temperature above which porosity begins to decrease (Marsh and Rodríguez-Reinoso 2006). In the case of the organic xerogels, the effect of temperature is clearly seen in Fig. 4, showing a correlation between the main textural parameters and the activating conditions. As a general rule, rising the temperature of activation resulted in higher surface areas and micropore volumes, regardless the impregnation method and the activating agent. The effect is more pronounced for the activation in K_2_CO_3_ by wet impregnation, as inferred from the straight line with a positive slope in the dependence of the specific surface area and micropore volume vs temperature. A similar trend can be seen for the narrow microporosity (Fig. S5). As an example, sample OG7-KC-W adsorbs about 1.7 and 6.7 times more carbon dioxide than OG7-KC-P and OG, respectively (Fig. S5). It is also important to highlight that the yields after activation (Table 2) were higher at 700 °C regardless the experimental conditions, indicating that the preservation of the mesoporosity of the pristine gel is not directly related with the consumption of the carbon matrix.

Regarding mesoporosity, large differences can be seen concerning both the mesopore volume (Table 2; Fig. 4) and the presence and position of the hysteresis loop in the N_2_ adsorption isotherms (Fig. 3). For the activation in K_2_CO_3_, the mesoporosity of the pristine xerogel is somewhat preserved at all temperatures, being the effect more pronounced for the samples obtained by wet impregnation (the dependence of the mesopore volume with the activation temperature followed a flat line). While the mesopore volume remains constant, the shape and position of the hysteresis loop changed significantly (Fig. 3). With the exception of sample OG7-KC-P, all the samples displayed prominent H2(a) hysteresis loops expanding over a wide range of relative pressures (from 0.45 to 0.9) and showing a steep decrease in the desorption branch at relative pressures close to 0.45. This is characteristic of pore systems formed by mesopores of different widths (wide openings), likely interconnected with narrow pore necks (Thommes et al. 2015). What is important to highlight is that the activation using K_2_CO_3_ enabled to preserve the mesoporosity of the pristine xerogel. When physical mixing is applied, the activation reaction is less efficient than wet impregnation, with lower surface areas and pore volumes (both micro- and mesopore) for the same temperature (Fig. 4).

For the activation with KOH by wet impregnation, an increment of the activation temperature results in a gradual development of microporosity. The shape and position of the hysteresis loops is similar to that described for the series prepared using K_2_CO_3_, indicating that the activation mechanism is likely similar for both chemicals. Differences affect mainly the mesopore volume (Table 2); the materials prepared using KOH and wet impregnation displayed the highest mesopore volumes, particularly for the lowest activation temperature. At 800 °C, the mesoporosity is no longer preserved regardless the impregnation procedure; this suggests that the reaction proceed through the rearrangement of the voids between the nodules that are responsible for the mesoporosity of the samples. The narrow microporosity was almost unaffected by the impregnation method, with the exception of materials prepared using carbonate at 700 °C, with similar pore volumes for series P and W (Fig. S5).

The differences in porosity of the samples depending on the chemical activating agents and impregnation procedures explain the discrepancies with the data reported in the literature (Calvo et al. 2011; Hayashi et al. 2002). For instance, Calvo et al. reported that the chemical activation using KOH of organic xerogels favors the formation of predominantly microporous materials that hardly inherit the mesoporosity of the organic precursor. We herein demonstrate that the porous features upon KOH activation of organic xerogels are heavily dependent on the activating conditions, with high temperatures leading to the destruction of the mesoporosity of the precursor. On the other hand, Hayashi et al. reported the chemical activation with K_2_CO_3_ of a phenol–formaldehyde resin, obtaining also mainly microporous carbons. In this case, the authors incorporated the K_2_CO_3_ during the polycondensation reaction of the phenol–formaldehyde precursors at 95 °C for 5 h. Since the porosity of the pristine resin is not reported, it is not possible to evaluate the impact of their procedure on the mesopore structure.

The impregnation method has also an important effect on the porosity of the material, especially at 700 °C; at this temperature the wet impregnation allows the development of the mesoporosity as opposed to physical mixing. This might be related to the more efficient contact between the activating agent and the precursor in the wet impregnation in an aqueous solution, as the organic xerogel is highly hydrophilic (ca. surface pH of 3.9 for sample OG), as also reported for similar materials (Issacs-Paez et al. 2015; Seredych et al. 2016).

**Fig. 5:**
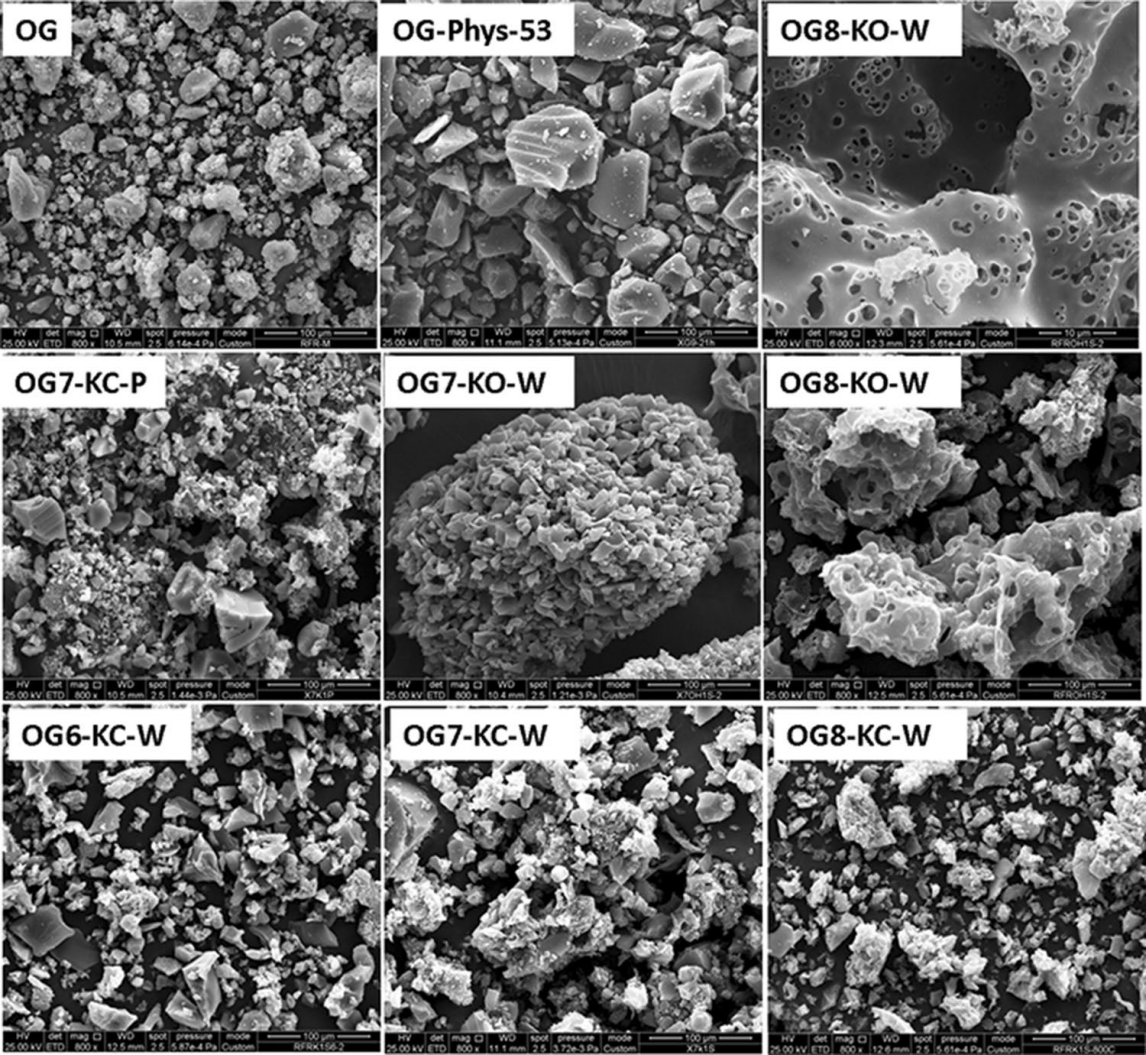
SEM images of the pristine organic xerogel and the carbon materials obtained by different activation methods and conditions

Figure 5 shows SEM images of the materials prepared by the different activation procedures (see other magnifications in Fig. S6). The chemical activation modified the morphology of the particles, leading to the formation of aggregates of varied sizes. This effect is more pronounced as the temperature of activation is increased, and for KOH than for K_2_CO_3_ -with little effect of the impregnation procedure-. In contrast, the physical activation (which was also carried out at a high activation temperature) barely modified the particle morphology of the carbon precursor.

To understand the differences in the development of porosity with the activation conditions, we must take into account the mechanism of activation for K_2_CO_3_ and KOH and the reactivity of the organic resin precursor, as the exact nature of the reactions and mechanisms involved during activation also depend on the nature of the carbon precursor (Wang and Kaskel 2012).

In the case of KOH, it is generally accepted that the porous development involves several reactions including the carbon lattice expansion by the intercalation of metallic potassium and the carbon gasification with H_2_O and CO_2_. KOH activation starts at temperatures above 400 °C with a dehydration reaction and formation of potassium compounds (typically K_2_CO_3_ and K_2_O) and other gases (i.e., H_2_, H_2_O, CO, CO_2_) commonly used as activating agents themselves (Wang and Kaskel 2012; Marsh and Rodríguez-Reinoso 2006). At temperatures higher than 700 °C, the as-formed K_2_CO_3_ and K_2_O are reduced by the carbon matrix or hydrogen to produce metallic potassium, whereas the K_2_CO_3_ may further decompose into CO_2_ and K_2_O.

In the case of activation with K_2_CO_3_ the reaction starts at around 800 °C; as a result, a lower consumption of the carbon matrix is expected, obtaining materials with a lower porous development (Mestre et al. 2015). Our data has shown that even when the activation temperature does not exceed 700 °C (series OG6), the activation occurs to some extent for KOH and K_2_CO_3_. Under these conditions metallic potassium is not formed, and hence the intercalation reaction does not occur. Therefore it can be inferred that the large mesopore volumes are obtained when the intercalation of metallic potassium does not take place. Furthermore, the thermal decomposition of the organic xerogel itself at 600 °C liberates CO_2_ and other light gases (Fig. S1) which are expected to trigger gasification reactions responsible for the formation of micropores and enlargement of mesopores. At 700 and 800 °C, the contribution of the activation due to the intercalation of potassium starts to be important (particularly at high temperatures where the mobility of metallic potassium by diffusion is favored), as well as the carbon consumption due to the transformation reactions of K_2_O and K_2_CO_3_. Consequently, the formation of metallic potassium would have a large impact on the yield and the mesoporosity of the resulting carbons, regardless the impregnation method.

## Conclusions

We report the synthesis of highly micro- and mesoporous carbon xerogels by the chemical activation of organic xerogels prepared by subcritical drying. An adequate choice of the activation conditions (i.e. type activating agent, impregnation method, activation temperature) allows preventing the structural collapse of the mechanically fragile resins commonly observed during conventional activation, rendering materials with a unique combination of micro-and mesopores at low activation temperatures.

The chemical activation in KOH and K_2_CO_3_ of the xerogel occurred at relatively low temperatures (i.e., 600 °C), due to the high reactivity of the precursor. Activation in K_2_CO_3_ rendered carbons with a well-developed microporosity and high mesopore volumes, partially inherited from the mesoporosity of the pristine xerogel. In the case of KOH activation below 800 °C, similar porous features (i.e., high micro and mesopore volumes) were obtained. At higher temperatures, KOH activation is more efficient (in terms of carbon consumption, and high surface areas and pore volumes) but the mesoporosity is completely destroyed. Furthermore, the impregnation method was found to have an important effect on the porosity final material. The incorporation of the activating agent by wet impregnation from aqueous solution allowed a more efficient contact with the resin, due to the high hydrophilic character of this precursor.

Overall, the yield and the mesoporosity of the carbons do not seem to be not directly related with the matrix consumption, but to the formation of metallic potassium. The large mesoporous structure is observed at low activating temperatures (i.e., 600 °C) when metallic potassium is not formed and hence the intercalation reaction within the carbon lattice does not take place. At higher temperatures, the contribution of the activation due to the intercalation of potassium becomes more important, and the obtained carbons present lower mesopore volumes (being the effect more pronounced in the case of KOH).

## Electronic supplementary material

Below is the link to the electronic supplementary material.

Supplementary material 1 (DOCX 8249 KB)
