# Supplementary material for: Designing micro- and mesoporous carbon networks by chemical activation of organic resins
Source: Adsorption (Boston). 2016 Dec 15;23(2):303–12. doi: 10.1007/s10450-016-9851-4 (PMC7064042; doi:10.1007/s10450-016-9851-4)
Supplement: Supplementary file 1 — Supplementary material 1 (DOCX 8249 KB) [file 10450_2016_9851_MOESM1_ESM.docx]

**Designing micro- and mesoporous carbon networks by chemical activation of organic resins**

Alicia Gomis-Berenguer^1^, Raquel García-González^1^, Ana S. Mestre^2^, Conchi O. Ania^1*^

^1^ADPOR Group, Instituto Nacional del Carbon (INCAR, CSIC), 33011 Oviedo (Spain)

^2^Centro de Química e Bioquímica, Faculdade de Ciências, Universidade de Lisboa, Portugal

**ELECTRONIC SUPPLEMENTARY MATERIAL**

**Fig. S1** TPD-MS of the pristine xerogel precursor (OG sample)

**Fig. S2** Derivative PSD curves calculated from N_2_ isotherms using 2D-NLDFT-HS method

**Fig. S3** High resolution nitrogen adsorption (solid symbols) and desorption (open symbols) isotherms at -196 °C of the pristine resin and the carbon xerogels after activation using K_2_CO_3_ and KOH at various temperatures and both impregnation methods (P and W).

**Fig. S4** Reactivity profiles of the mixture xerogel precursor (sample OG) with KOH and K_2_CO_3_ for both impregnation methods (physical mixing, P, and wet impregnation, W). Experimental conditions for the thermogravimetric profiles: under N_2_ (50 mL/min) up to 900 °C (ramp 15 °C/min)

**Fig. S5** Adsorption isotherms of carbon dioxide at 0 °C of the xerogels activated (A-C-E) with K_2_CO_3_ and (B-D-F) with KOH by wet impregnation (W) and physical mixing (P).

**Fig. S6** SEM images of the pristine xerogel (sample OG) and the carbon materials obtained by different activation methods and conditions.

**Fig. S1** TPD-MS of the pristine xerogel precursor (OG sample)

**Fig. S2** Derivative PSD curves calculated from N2 isotherms using 2D-NLDFT-HS method

**Fig. S3** High resolution nitrogen adsorption (solid symbols) and desorption (open symbols) isotherms at -196 °C of the pristine resin and the carbon xerogels after activation using K_2_CO_3_ and KOH at various temperatures and both impregnation methods (P and W)

**Fig. S4** Reactivity profiles of the mixture xerogel precursor (sample OG) with KOH and K_2_CO_3_ for both impregnation methods (physical mixing, P, and wet impregnation, W). Experimental conditions for the thermogravimetric profiles: under N_2_ (50 mL/min) up to 900 °C (ramp 15 °C/min)

**Fig. S5** Adsorption isotherms of carbon dioxide at 0 °C of the xerogels activated (A-C-E) with K_2_CO_3_ and (B-D-F) with KOH by wet impregnation (W) and physical mixing (P).


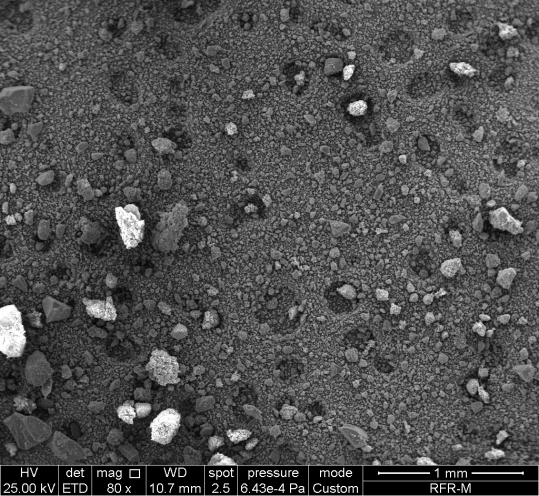

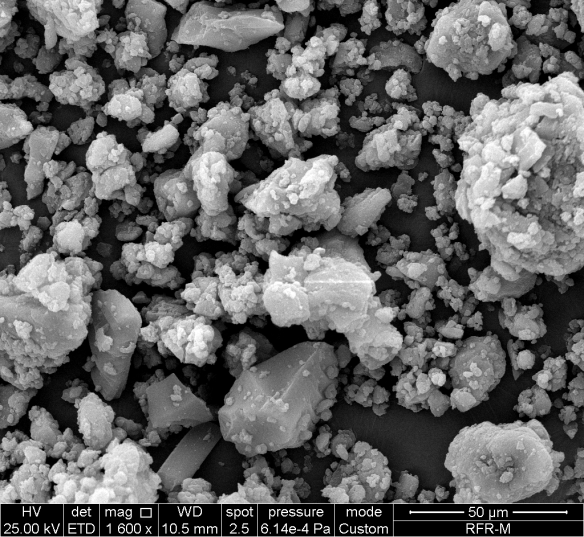

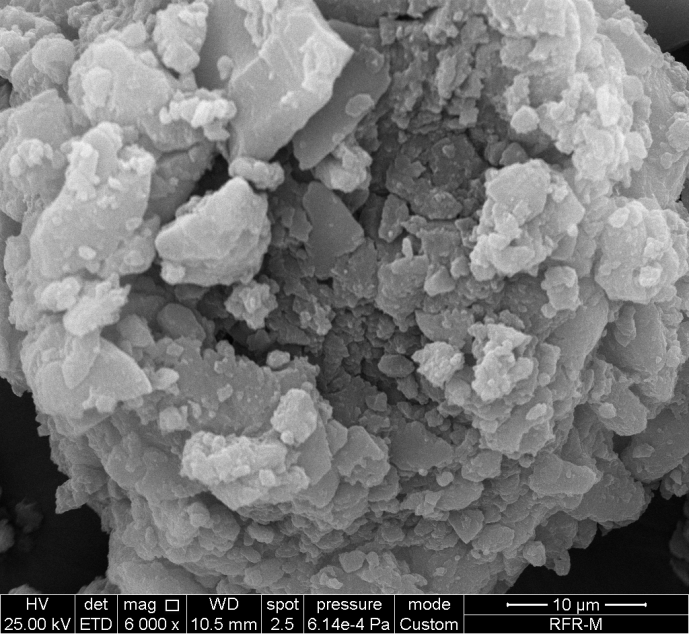


**OG**


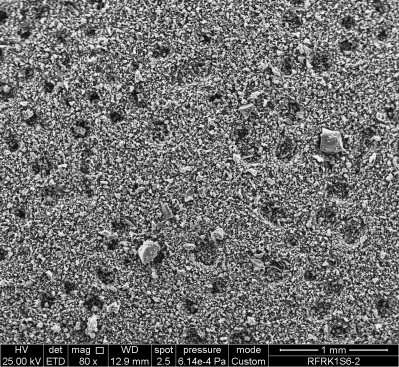

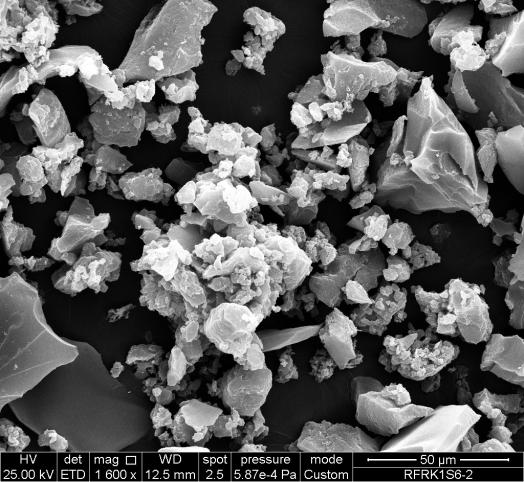

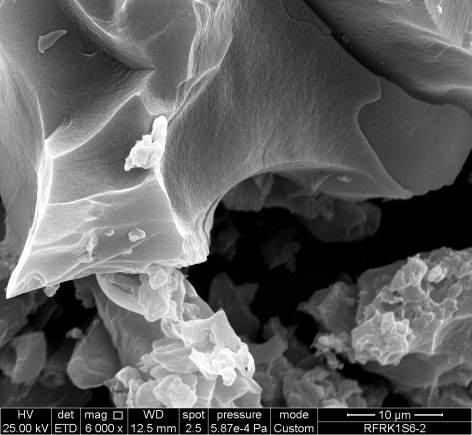


**OG6-KC-W**


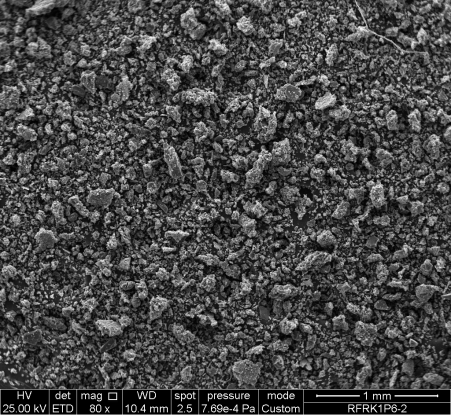

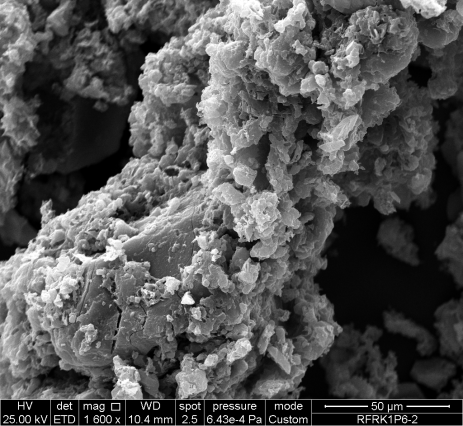

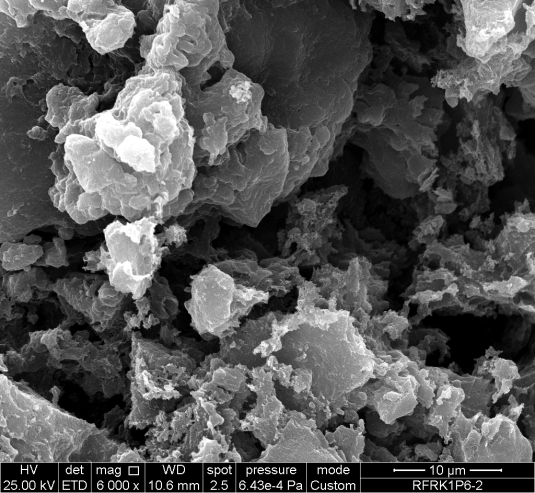


**OGG-KC-P**

**Fig. S6** SEM images of the pristine xerogel (sample OG) and the carbon materials obtained by different activation methods and conditions.


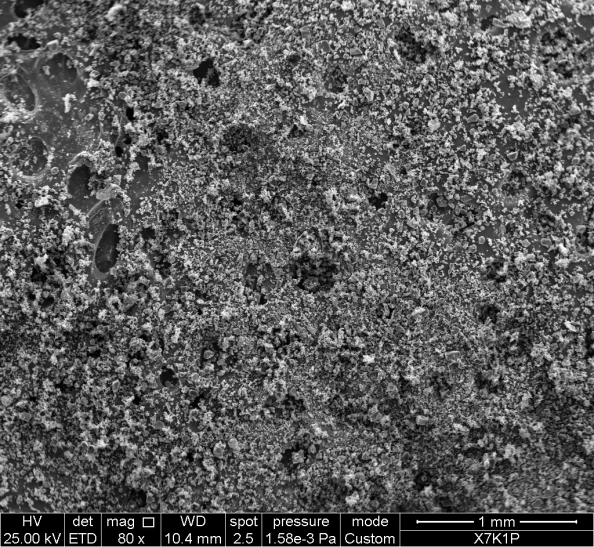

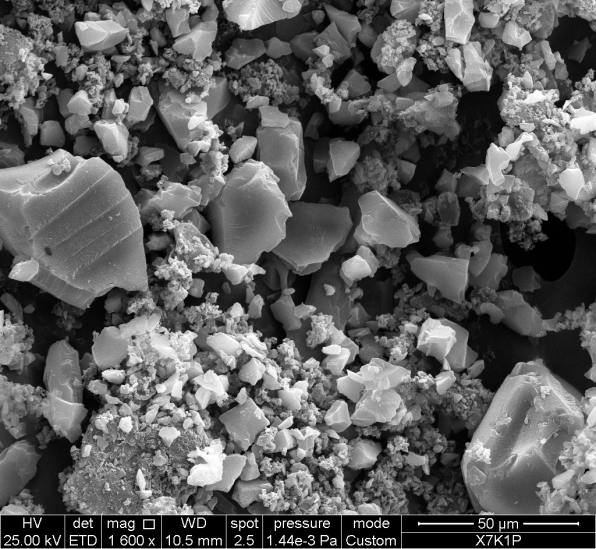

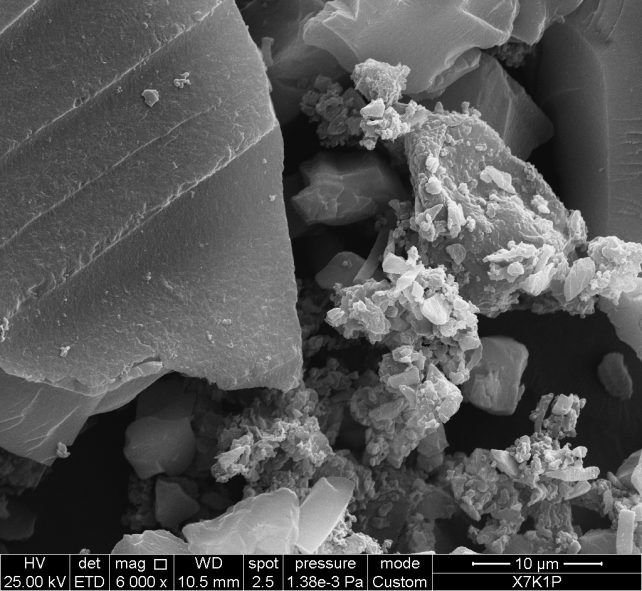


**OG7-KC-P**


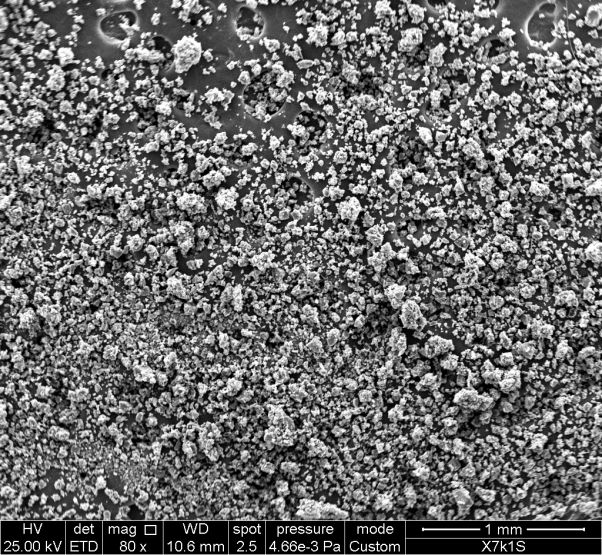

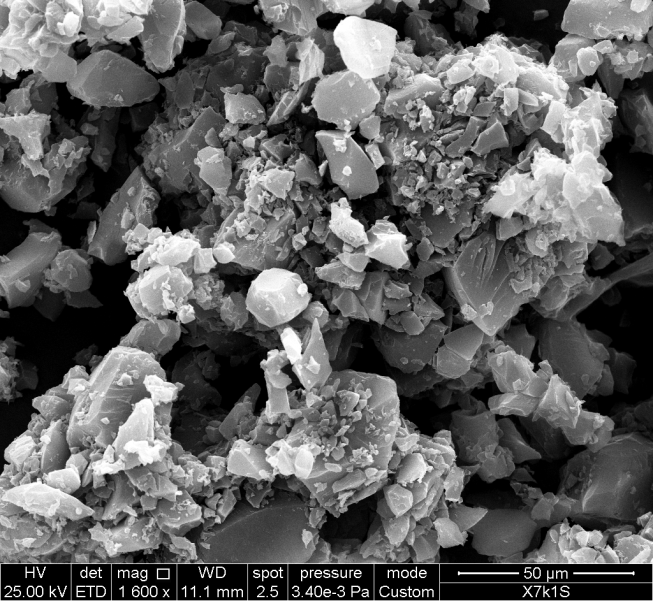

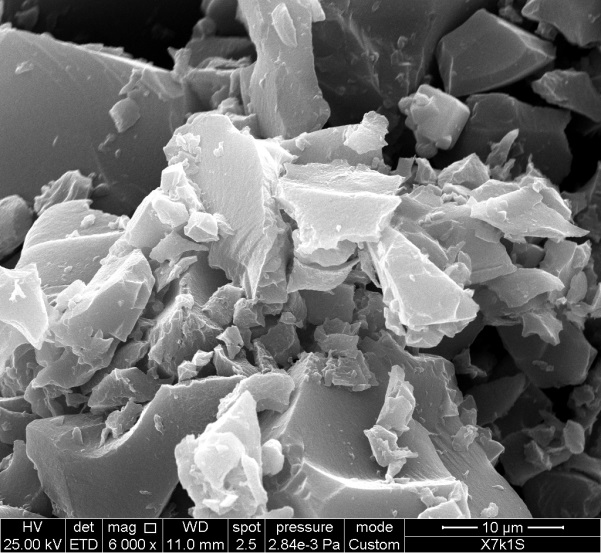


**OG7-KC-W**


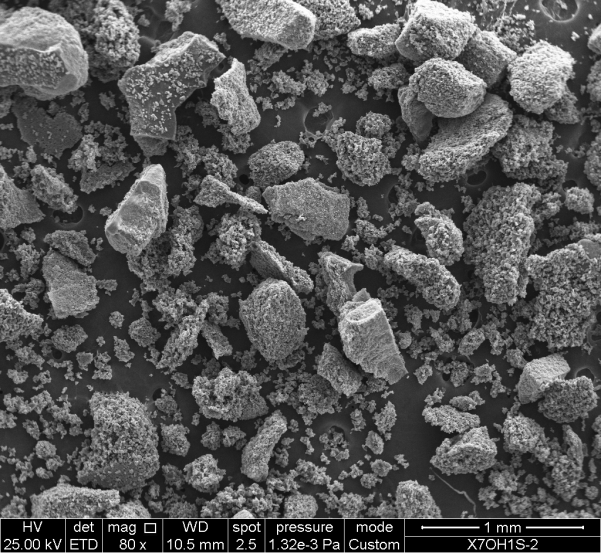

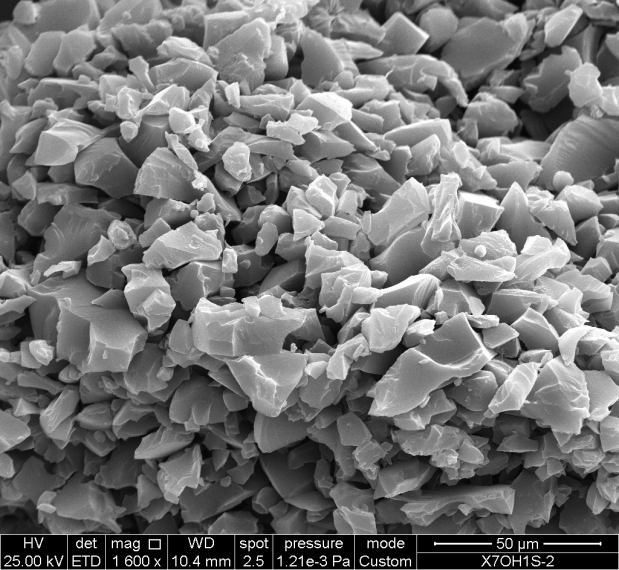

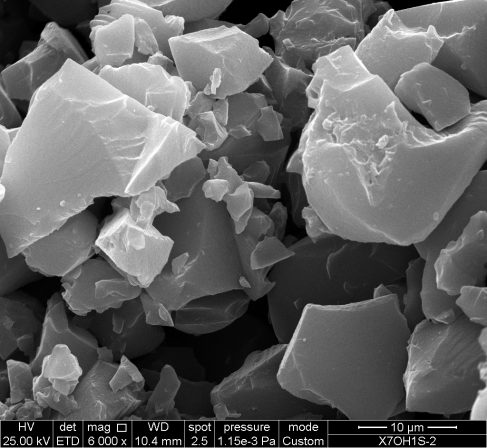


**OG7-OH-W**


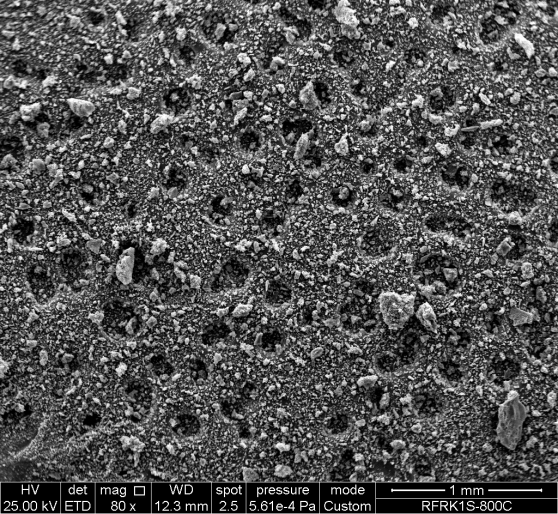

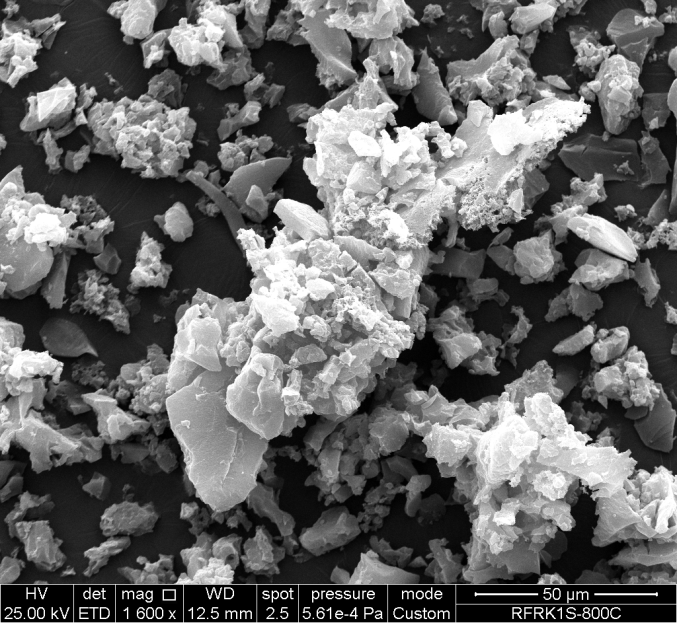

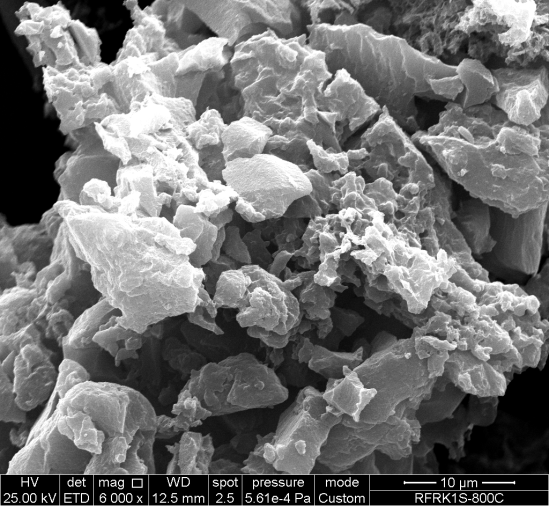


**OG8-KC-W**


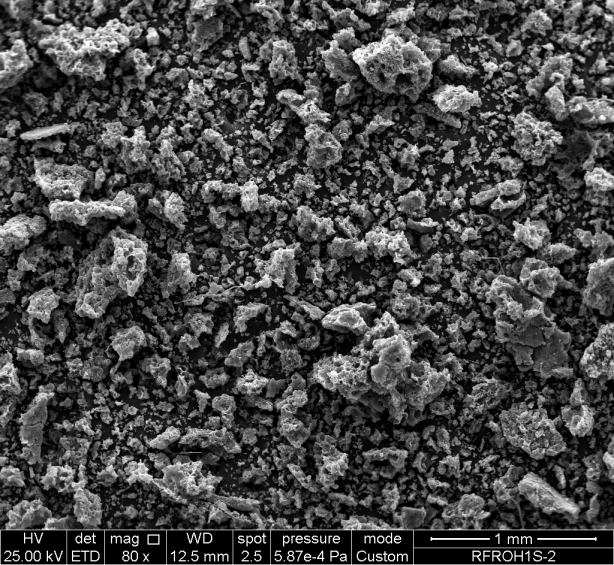

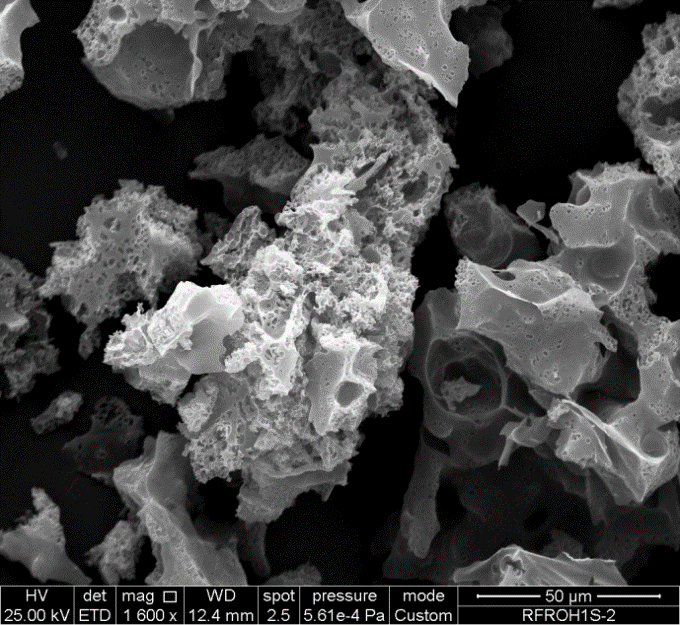

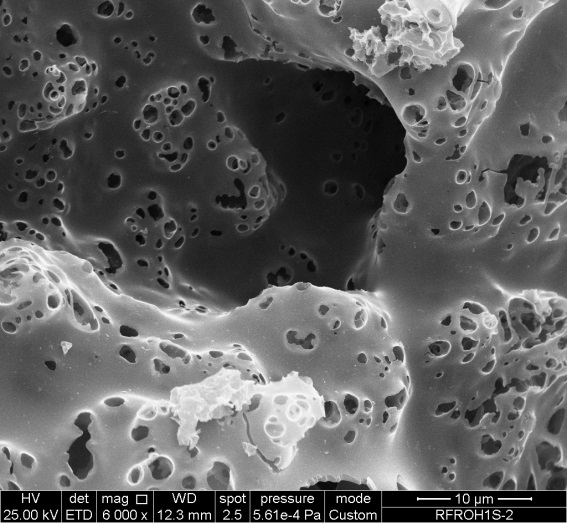


**OG8-OH-W**

**Fig. S6** (cont) SEM images of the pristine xerogel (sample OG) and the carbon materials obtained by different activation methods and conditions.
